# Exploratory analyses of leukocyte responses in hospitalized patients treated with ozanimod following a severe acute respiratory syndrome coronavirus 2 (SARS‑CoV‑2) infection

**DOI:** 10.1111/imcb.70006

**Published:** 2025-03-02

**Authors:** Olivier Courtemanche, Pascale Blais‐Lecours, Sylvie Lesage, Geneviève Chabot‐Roy, Lise Coderre, Marie‐Renée Blanchet, Nathalie Châteauvert, François Lellouche, David Marsolais

**Affiliations:** ^1^ Centre de recherche de l'Institut Universitaire de Cardiologie et de Pneumologie de Québec ‐ Université Laval Québec QC Canada; ^2^ Centre de Recherche de l'Hôpital Maisonneuve‐Rosemont Montréal QC Canada; ^3^ Département de microbiologie, infectiologie et immunologie Université de Montréal Montreal QC Canada; ^4^ Département de Médecine Université Laval Quebec QC Canada

**Keywords:** anti–SARS‐CoV‐2 antibodies, clinical trial, COVID‐19, monocytes, S1P

## Abstract

Sphingosine‐1‐phosphate receptor 1 (S1P_1_) ligands effectively reduce immunopathological damage in viral pneumonia models. Specifically, S1P_1_ ligands inhibit cytokine storm and help preserve lung endothelial barrier integrity. We recently showed that the S1P receptor ligand ozanimod can be safely administered to hospitalized patients with coronavirus disease 2019 (COVID‐19) exhibiting severe symptoms of viral pneumonia, with potential clinical benefits. Here, we extend on this study and investigate the impact of ozanimod on key features of the immune response in patients with severe COVID‐19. We quantified circulating cytokine levels, peripheral immune cell numbers, proportions and activation status; we also monitored the quality of the humoral response by assessing anti–severe acute respiratory syndrome coronavirus 2 (SARS‑CoV‑2) antibodies. Our findings reveal that patients receiving ozanimod during acute SARS‐CoV‐2 infection exhibit significantly reduced numbers of circulating monocytes compared with those receiving standard care. Correspondingly, in the ozanimod‐treated group, circulating levels of C–C motif ligand 2 (CCL2) were decreased. While treatment with ozanimod negatively impacted the humoral response to COVID‐19 in unvaccinated patients, it did not impair the development of a robust anti–SARS‐CoV‐2 antibody response in vaccinated patients. These findings suggest that ozanimod influences key immune mechanisms during the acute phase of SARS‐CoV‐2 infection.

## Graphical abstract

In this exploratory study on the putative mechanisms of action of ozanimod in the modulation of severe coronavirus disease 2019 (COVID‐19), we found that patients receiving standard of care plus ozanimod had reduced circulating monocytes and C–C motif ligand 2 (CCL2) levels, compared with patients receiving standard of care alone. Moreover, while ozanimod appeared to mitigate the humoral response in unvaccinated patients, it did not hinder the development of a strong anti–severe acute respiratory syndrome coronavirus 2 (SARS‑CoV‑2) antibody in vaccinated individuals.

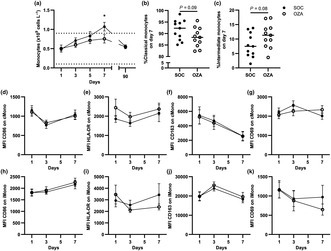

## INTRODUCTION

Coronavirus disease 2019 (COVID‐19) is caused by the highly contagious severe acute respiratory syndrome coronavirus 2 (SARS‑CoV‑2). Even with the advent of effective vaccines and antiviral therapy, breakthrough cases still occur, some leading to serious life‐threatening infections. Severe COVID‐19 is characterized by endothelial overactivation and dysfunction, high levels of local and systemic inflammatory mediators, persistent recruitment of immune cells to the lungs and leucocyte dysfunction.^1^, ^2^, ^3^

Sphingosine‐1‐phosphate (S1P) is a bioactive lipid that activates five receptors (S1P_1_–S1P_5_) with important roles in angiogenesis, inflammation, cell migration and vascular barrier integrity. Low levels of S1P have been associated with COVID‐19 severity, hospital admission and death,^4^, ^5^ supporting S1P signaling as a potential target in severe COVID‐19. Further supporting this, preclinical models of severe viral pneumonia and pulmonary inflammation have shown that S1P signaling through S1P_1_ can reduce inflammation by repressing the over‐recruitment of immune cells, interfering with specific cytokine release, including C–C motif ligand 2 (CCL2) and interleukin (IL)‐6, and maintaining the lung endothelial barrier integrity.^6^, ^7^, ^8^

Ozanimod is one of the S1P receptor–targeting disease‐modifying drugs presently used in multiple sclerosis and ulcerative colitis. As with other synthetic S1P receptor ligands, ozanimod decreases peripheral lymphocyte levels by limiting their egress from lymph nodes and controlling endothelial integrity. We recently showed that ozanimod could efficiently modulate human B‐cell activation and lower their cytokine production *ex vivo* in response to an inflammatory stimulus.^9^ In severe COVID‐19, patients with multiple sclerosis under ozanimod therapy may have decreased SARS‐CoV‐2–specific antibody levels and altered SARS‐CoV‐2–specific T‐cell response compared with untreated patients.^10^, ^11^ However, animal studies using S1P receptor ligands show that they do not significantly impair the humoral response and pathogen neutralization when administered after the onset of a viral infection.^6^, ^12^

As for treatment beginning during acute SARS‐CoV‐2 infection, we, as well as others, showed that S1P receptor ligands can safely be administered to patients hospitalized for viral pneumonia, with potential clinical benefits.^13^, ^14^ However, questions remain about the potential mechanisms of action of S1P receptor ligands in humans with severe and acute viral infections and whether these mechanisms align with those observed in animal models. Here, through the COVID‐19 Intervention Study (COZI) trial,^14^ we performed exploratory analyses to study the impact of ozanimod on key features of the immune response in severe COVID‐19 cases. Circulating cytokine levels, peripheral immune cells and the specific SARS‐CoV‐2 humoral response were longitudinally monitored in patients receiving the standard of care (SOC) group or the SOC plus ozanimod (OZA) group. No significant differences were observed in the general composition of the immune system between the two treatment groups at baseline. The OZA group exhibited a significantly decreased number of circulating monocytes and also displayed lower circulating CCL2 levels. Given the pilot nature of the trial, we also report trends for future mechanistic investigations. For instance, IL‐8 levels appeared to be decreased in the OZA group, compared with SOC. Moreover, unvaccinated patients receiving ozanimod tended to have low levels of anti–SARS‐CoV‐2 antibodies on day 90 compared with the SOC group, a limitation that was overcome in vaccinated individuals.

## RESULTS

### Ozanimod does not modify the proportion and overall activation phenotype of circulating lymphocytes

**Figure 1:**
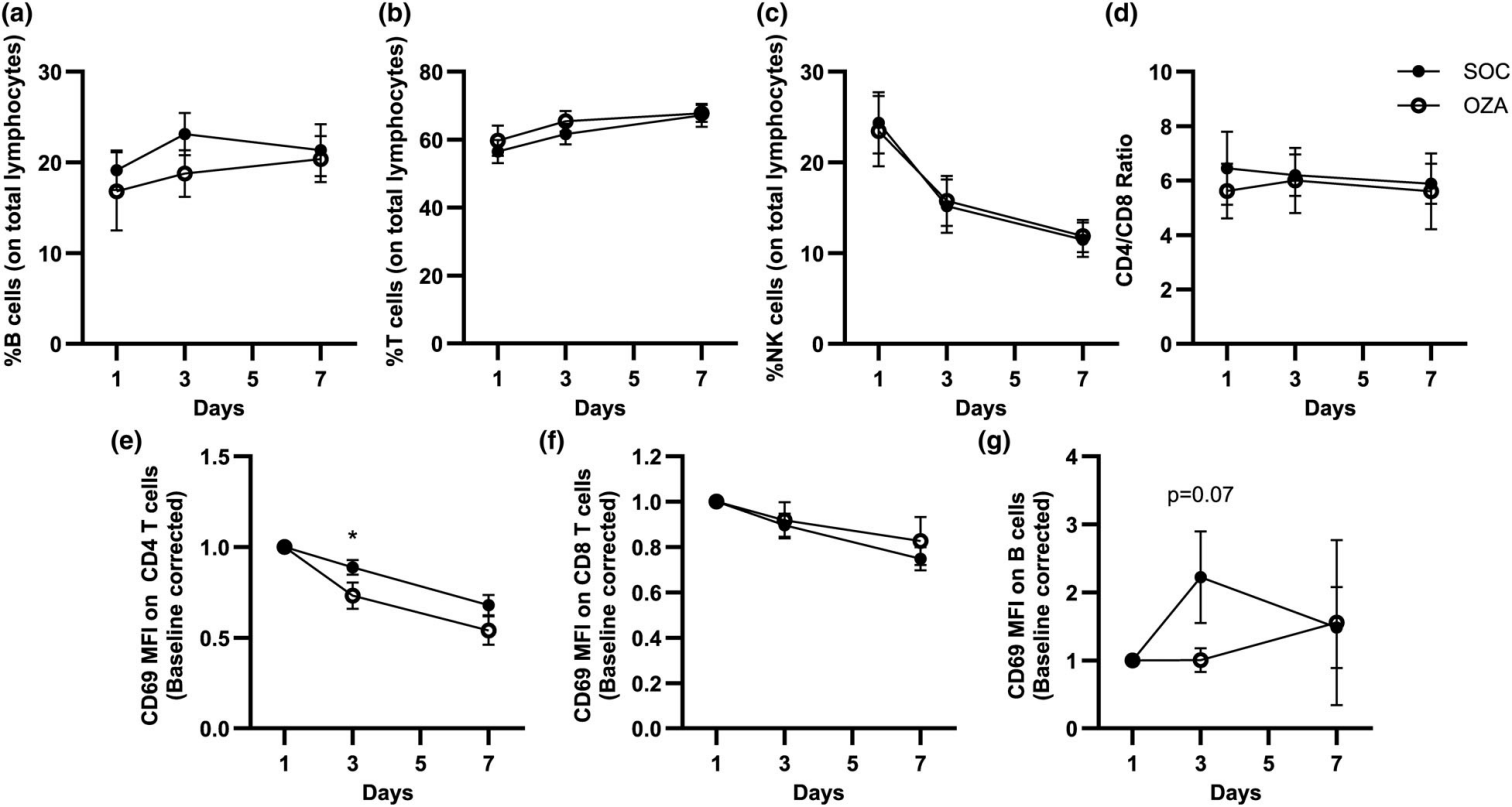
Impact of ozanimod on circulating lymphocyte proportions and CD69 surface expression in patients with coronavirus disease 2019 (COVID‐19). Peripheral blood mononuclear cells (PBMCs) from patients with severe COVID‐19 were isolated for flow cytometry analyses. Gating was performed as shown in Supplementary figure 1a and the proportion of **(a)** B cells, **(b)** T cells and **(c)** natural killer (NK) cells were quantified in both standard of care (SOC) and ozanimod (OZA) groups. **(d)** CD4 and CD8 T cells are shown as a ratio. Median fluorescence intensities (MFIs) for CD69 on **(e)** CD4 T cells, **(f)** CD8 T cells and **(g)** B cells are depicted. Baseline fluorescence using fluorescent minus one control was removed from each patient on each day. Each result was baseline‐corrected by dividing the MFI on each day by the MFI for the same patient on day 1. Averages ± standard errors of the mean are shown for each tested day for each group. *n* = 11–22 for SOC and 11–18 for OZA. *P < 0.05.

Patients with severe COVID‐19 show a sharp transient reduction in circulating lymphocyte numbers, which subsequently return to normal after a few days.^15^ We showed that this return to normal was delayed when ozanimod was administered during acute COVID‐19 infection in our cohort.^14^ However, the composition of lymphocytes between the SOC group and the OZA group remained undefined. To fill this knowledge gap, we quantified T‐, B‐ and natural killer (NK) cell proportions by flow cytometry (see Supplementary figure 1 for the gating strategy). For both the SOC and OZA groups, the proportion of B cells, T cells and NK cells were similar at all time points tested, namely, on day 1 (baseline, before ozanimod), day 3 and day 7 (Figure 1a–c). Notably, in both groups of patients, the proportion of B (Figure 1a) and T cells (Figure 1b) slightly increased between days 1 and 7, while the proportion of NK cells steeply declined (Figure 1c). Even if the proportion of T cells increased over time, the CD4/CD8 T‐cell ratio remained stable throughout the time course, for both patient groups (Figure 1d). The proportion of effector, naïve, central memory and terminally differentiated T cells expressing CD45RA did not significantly differ between the patient groups (Supplementary figure 2). These results indicate that, while patients in the OZA group had a lower circulating lymphocyte count,^14^ the general lymphocyte subset distribution remained similar in both experimental arms of this cohort.

CD69 is an early lymphocyte activation marker, which is implicated in peripheral lymphocyte retention.^16^, ^17^ S1P_1_ and CD69 are mutual regulators,^18^ and S1P_1_ ligands reduce CD69 levels in B cells *in vitro*.^9^ Yet, evidence of different S1P_1_ signaling mechanisms exists in B cells, CD4 and CD8 T cells.^19^ As such, we measured the impact of ozanimod on CD69 levels in the different lymphocyte subsets. In line with the role of S1P_1_ ligands in reducing CD69 levels on CD4 T cells,^20^ CD69 median fluorescence intensity was significantly reduced on day 3 on CD4 T cells from the OZA group relative to the SOC group (Figure 1e). By contrast, no differences were observed regarding CD69 levels on CD8 T cells between both groups at all time points examined (Figure 1f). In B cells, the increase in CD69 observed at day 3 compared with baseline appeared to be lower in the OZA group compared with the SOC (*P* = 0.07; Figure 1g). Other lymphocyte activation markers such as programmed cell death protein 1 and the proportion of human leukocyte antigen‐DR^+^CD38^+^ T cells did not differ between the SOC and OZA groups (Supplementary figure 2). This suggests that ozanimod did not modify the overall activation profile of circulating lymphocytes.

### Ozanimod modifies monocyte levels during severe COVID‐19

**Figure 2:**
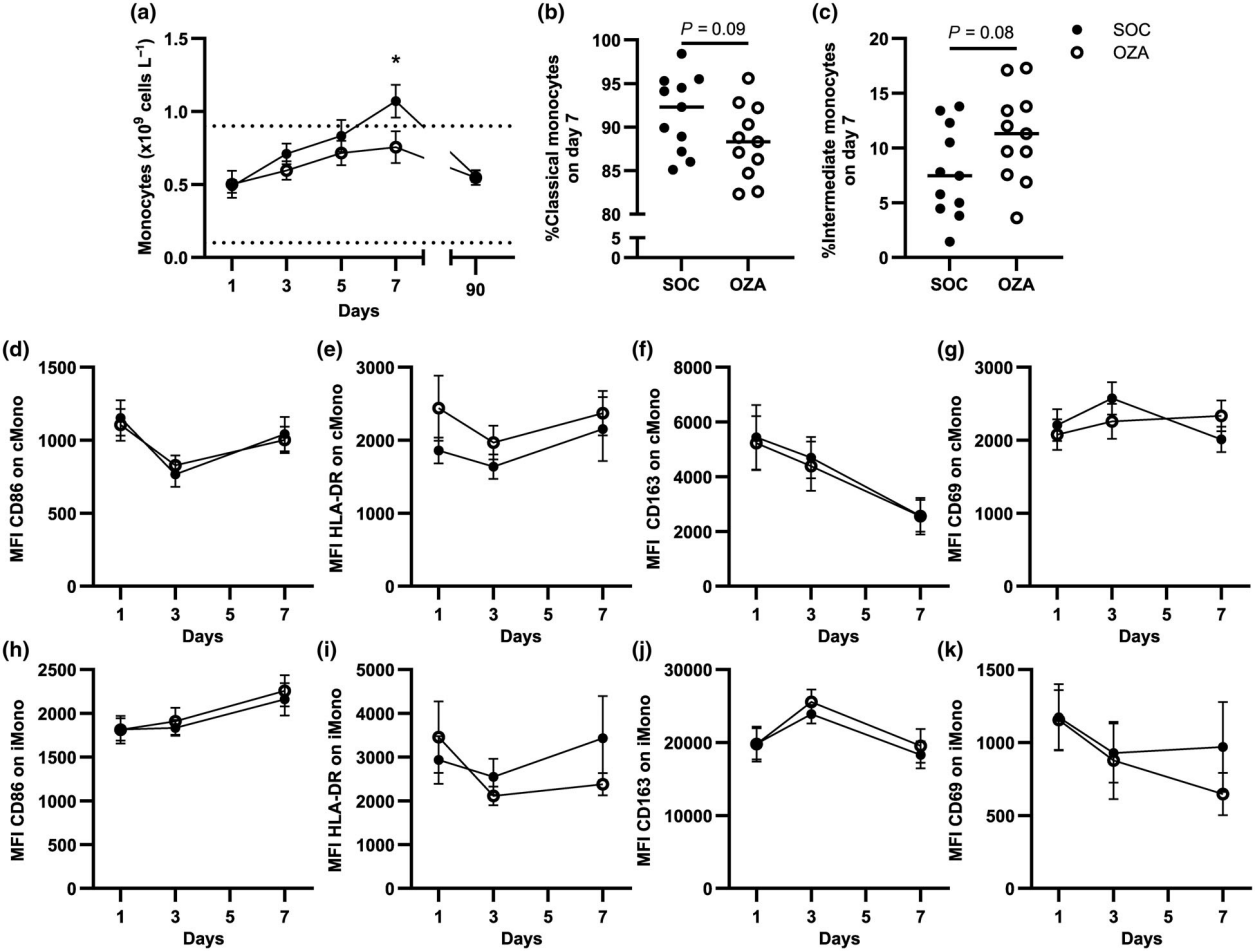
Circulating monocyte levels are lower in the ozanimod (OZA) group compared with the standard of care (SOC) group. **(a)** Complete blood counts were performed to assess total circulating monocytes. **(b–k)** Peripheral blood mononuclear cells (PBMCs) from patients with severe coronavirus disease 2019 (COVID‐19) were isolated and monocytes were analyzed by flow cytometry. Gating was performed as described in Supplementary figure 1b. **(b)** Classical monocyte and **(c)** intermediate monocyte frequencies were computed at day 7, that is, when significant differences were observed between both groups for total monocyte numbers. Median fluorescence intensities (MFIs) on classical monocytes **(d–g)** and intermediate monocytes **(h–k)** were measured for CD86, human leukocyte antigen (HLA)‐DR, CD163 and CD69. Baseline fluorescence from fluorescence minus one control was removed for every patient. cMono, classical monocytes; iMono, intermediate monocytes. Averages ± standard errors of the mean are shown for each tested day for each group. *n* = 11–22 for SOC and 11–18 for OZA. *P < 0.05.

As seen for the number of circulating lymphocytes,^14^ complete blood cell counts showed a reduction of monocytes in the OZA group on day 7 (Figure 2a). During the first 7 days after enrollment, nonclassical monocytes were completely depleted from circulation in the OZA and SOC groups (not shown). The proportion of classical and intermediate monocytes was comparable for both groups at baseline (not shown). However, on day 7, classical monocytes appeared to be decreased in the OZA group relative to the SOC group (*P* = 0.09; Figure 2b), and the counterbalancing effect for a higher proportion of intermediate monocytes was also observed (*P* = 0.08; Figure 2c). These results suggest that the ozanimod‐associated decrease of circulating monocytes observed on day 7 may be associated with a modification of classical *versus* intermediate monocyte proportions.

COVID‐19 is linked to dysregulations in monocyte subsets in severe cases, with an elevation of activation markers, such as CD163 and CD69, and a downregulation of human leukocyte antigen‐DR.^21^ We observed no significant differences between the SOC and OZA groups regarding CD86, human leukocyte antigen‐DR, CD163 and CD69 surface expression (Figure 2d–k) in both circulating classical and intermediate monocyte populations. Together, these data suggest that ozanimod reduces circulating monocytes and may influence the balance between classical and intermediate monocytes while not altering their overall activation profile in the circulation.

### Ozanimod reduces circulating CCL2 levels during the acute phase of COVID‐19

**Figure 3:**
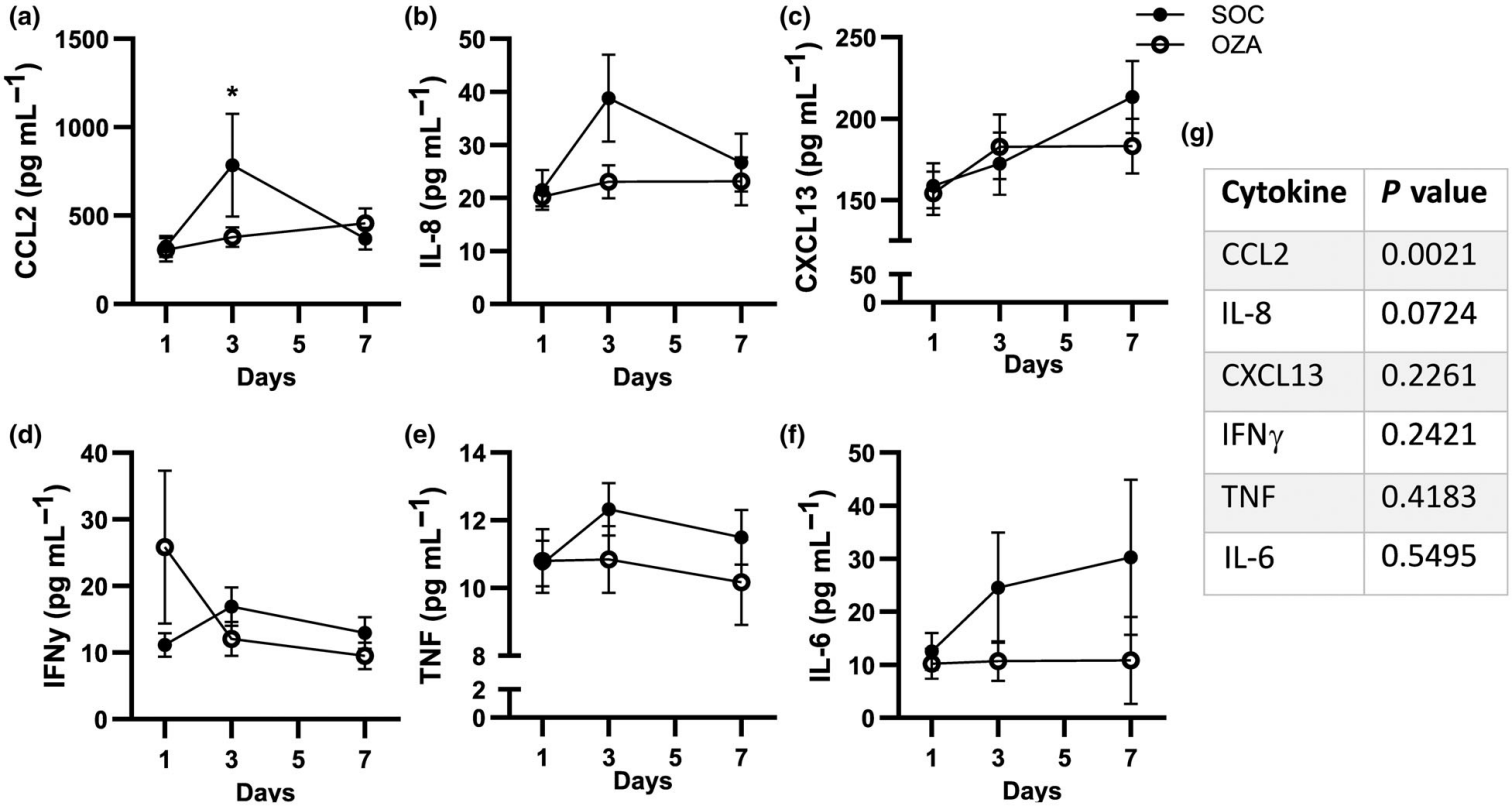
Longitudinal comparison of specific circulating cytokines between the standard of care (SOC) and ozanimod (OZA) groups. The serum sample was collected at baseline (day 1; before ozanimod administration) and during treatment (on days 3 and 7). **(a–f)** A Luminex assay (23‐plex; Bio‐Techne) was performed on serum to evaluate levels of various cytokines in both groups. **(g)** *P*‐values for the group–time interaction for each circulating mediator are indicated. Averages ± standard errors of the mean are shown for each tested day for each group. *n* = 11–22 for SOC and 11–18 for OZA. CCL2, C–C motif ligand 2; CXCL13, C–X–C motif ligand; IFN, interferon; IL, interleukin; TNF, tumor necrosis factor. *P < 0.05.

Patients with severe COVID‐19 show high levels of circulating cytokines, growth factors and adhesion molecules that correlate with disease severity.^1^, ^22^ As S1P_1_ modulators, such as ozanimod, are associated with a decrease of specific cytokines under inflammatory conditions,^6^, ^23^ we assessed the impact of ozanimod on key inflammatory mediators in patients with severe COVID‐19. First, we observed that the levels of cytokines classically associated with severe COVID‐19, including IL‐8, tumor necrosis factor and IL‐6, were found at previously documented levels^24^, ^25^ in our cohort (Figure 3b, e, f). Next, we observed that CCL2 was significantly decreased on day 3 in the OZA group compared with the SOC group (Figure 3a). IL‐8 also showed a trend to be reduced on day 3 in the OZA group (*P* = 0.07; Figure 3b) and *P‐*values are reported for other cytokines such as C–X–C motif ligand (CXCL) 13, interferon‐γ, tumor necrosis factor and IL‐6 (Figure 3c–f; see Supplementary figure 3 for the remaining analytes). These results align with the theory that the administration of ozanimod during the acute phase of a viral infection could limit the increase of specific circulating inflammatory cytokines.

**Figure 4:**
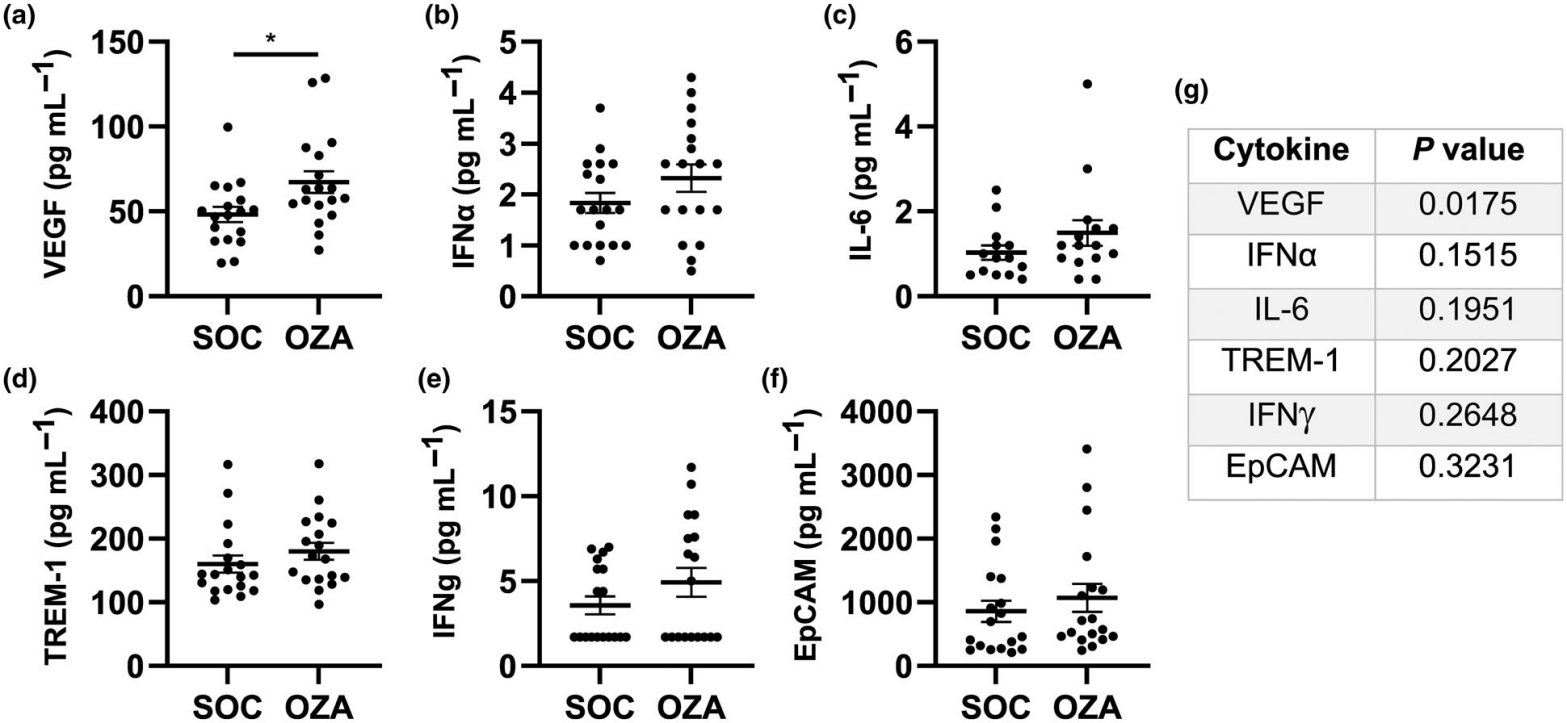
Circulating mediators at the follow‐up visit. The serum sample was collected on a follow‐up visit on day 90. **(a–f)** A Luminex assay was performed to evaluate the levels of various circulating mediators in both groups. **(g)** *P‐*values for each circulating mediator are indicated. Individual patient values (dots) and averages (horizontal line) ± standard errors of the mean are shown. *n* = 14–18 for the standard of care (SOC) group and 15–18 for the ozanimod (OZA) group. EpCAM, epithelial cellular adhesion molecule; IFN, interferon; IL, interleukin; TREM‐1, triggering receptors expressed on myeloid cells 1; VEGF, vascular endothelial growth factor. *P < 0.05.

In contrast to the reduced cytokine response during the acute phase of the disease, the OZA group appeared to have increased levels of circulating mediators compared with the SOC group at the 90‐day follow‐up. Specifically, vascular endothelial growth factor levels were significantly higher in the OZA group (Figure 4a), while statistical analyses for interferon‐α, IL‐6, triggering receptors expressed on myeloid cells 1 (TREM‐1), interferon‐γ and epithelial cellular adhesion molecule (EpCAM) suggested a potential pattern toward an increase (*P* = 0.1515–0.3231; Figure 4b–g; see Supplementary figure 4 for remaining analytes).

### Ozanimod likely impedes the antibody response to the primary infection

**Figure 5:**
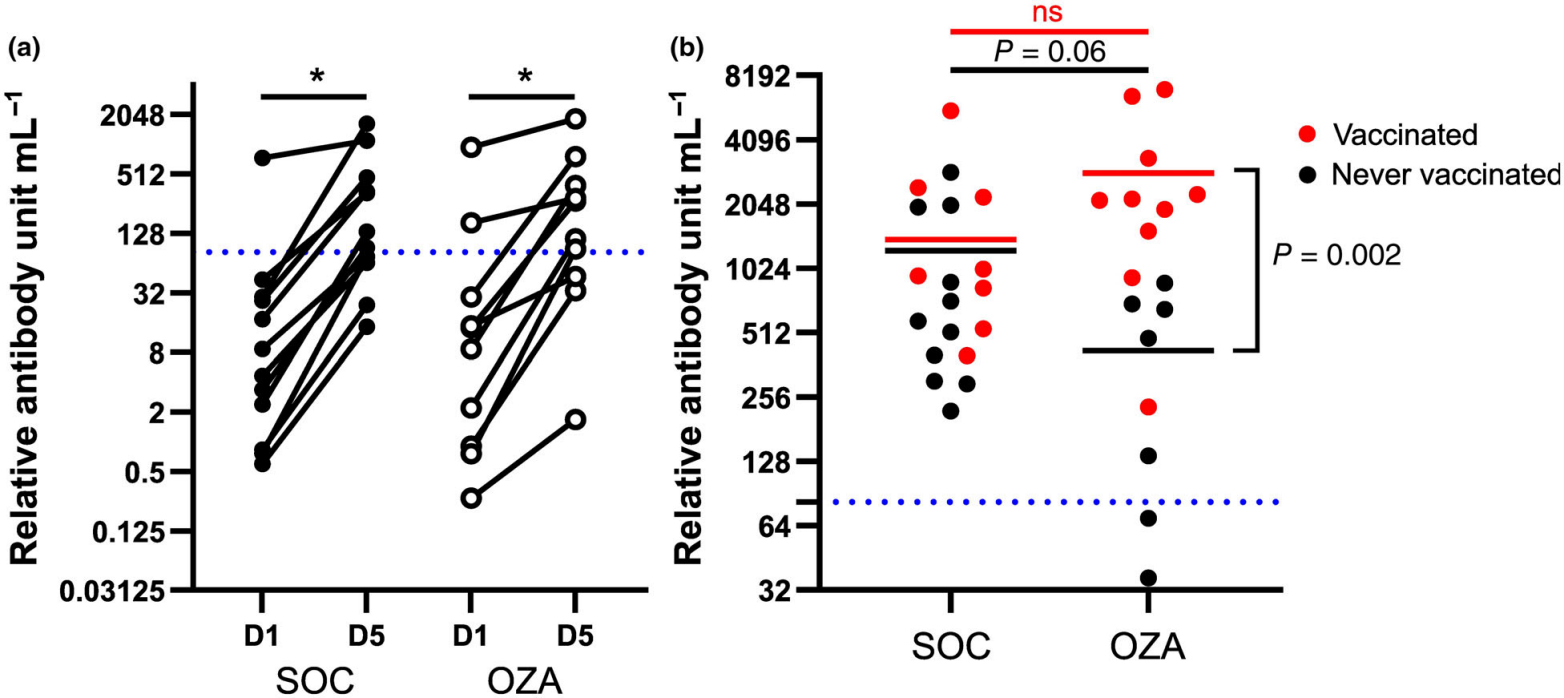
Ozanimod hinders the primary humoral response to severe acute respiratory syndrome coronavirus 2 (SARS‑CoV‑2). The serum sample was collected at baseline [day 1 (D1)] on day 5 (D5) and on a follow‐up visit on day 90 after hospital admission to measure the impact of ozanimod on the concentration of anti–SARS‐CoV‐2 immunoglobulin Gs. **(a)** Relative antibody‐level progression is shown for each eligible patient between D1 and D5 in the standard of care (SOC) and the ozanimod (OZA) groups. Paired *t*‐tests were used between D1 and D5 to assess changes in relative antibody unit mL^−1^ in both groups. **(b)** The mean for unvaccinated (black dots) and vaccinated (red dots) patients from each group is depicted with a horizontal line and the 80% protection threshold against symptomatic infection as shown by Feng *et al*.^26^ is delimited by a blue dashed line. 50% of patients received at least one vaccine shot before the follow‐up visit. *t*‐tests were performed to assess whether SARS‐CoV‐2–specific antibody levels were different between the two groups when vaccinated (red) and unvaccinated (black; *P* = 0.06). *n* = 19 (8 vaccinated) for the SOC group and *n* = 17 (10 vaccinated) for the OZA group. ns, not significant. *P < 0.05.

Multiple patients with sclerosis on S1P receptor modulator therapies show reduced SARS‐CoV‐2–specific antibody levels after infection and/or vaccination.^10^ In contrast to our cohort, these patients are already on S1P receptor ligand therapy at the onset of infection. We thus decided to determine whether the onset of ozanimod therapy during the acute phase of the disease impacted the antibody response. In our cohort, patients were recruited on average 10 days after the beginning of symptoms.^14^ In Figure 5a, only patients who were unvaccinated before study recruitment are shown. On study day 1, the vast majority of patients already had detectable levels of anti–SARS‐CoV‐2 immunoglobulin (Ig)Gs (Figure 5a). Between days 1 and 5, all patients showed an increase in their anti–SARS‐CoV‐2 IgG titers. However, on day 90, unvaccinated patients receiving ozanimod tended to display lower levels of anti–SARS‐CoV‐2 IgGs (*P* = 0.06) compared with the SOC group (Figure 5b). Besides, while all unvaccinated patients in the SOC group had higher anti–SARS‐CoV‐2 IgG levels than the 80% reinfection threshold, as determined by Feng *et al*.,^26^ two of the seven unvaccinated patients receiving ozanimod had levels below this threshold (Figure 5b). Patients vaccinated before the follow‐up visit in the OZA group all showed robust levels of anti–SARS‐CoV‐2 IgGs, which were significantly augmented compared with unvaccinated patients in that group. Importantly, vaccinated patients in the OZA group showed anti–SARS‐CoV‐2 IgG levels that were similar to that of vaccinated patients in the SOC group (Figure 5b). These results suggest that even though ozanimod does not prevent the increase of the antibody response in the acute phase of an ongoing immune response, its transient administration can reduce long‐term antibody levels. However, vaccination before or after the ozanimod transient therapy (see Supplementary table 2 for details) overcame this effect.

## DISCUSSION

In the last two decades, S1P_1_ modulators were shown to be effective in controlling lung inflammation in animal models of viral pneumonia by decreasing endothelial overactivation and increasing lung endothelial barrier integrity.^6^, ^7^, ^27^ More recently, S1P_1_ ligands were shown to be relatively safe in small cohorts of patients with acute SARS‐CoV‐2 infection, with potential clinical benefits such as reduced hospitalization time and diminished rehospitalization rate.^13^, ^14^ We set out to determine whether these clinical findings could be strengthened by investigating whether previously described or undescribed mechanisms of action were at play.

Similar to other S1P_1_ modulators, ozanimod leads to a profound decrease in circulating B and T cells in healthy volunteers and chronic disease patients, which is time and dose dependent.^28^, ^29^, ^30^ We previously showed that ozanimod administration to patients with COVID‐19 moderately lowered lymphocyte levels compared with SOC.^14^ Using flow cytometry, we show here that this moderate lymphocyte diminution was distributed between the different lymphocyte populations. Indeed, T‐, B‐ and NK cell proportions were not significantly affected by ozanimod, and the CD4‐to‐CD8 ratio was similar between both groups. No circulating CD4 or CD8 subpopulations, that is, naïve T cells, central memory T cells, effector memory T cells and effector memory cells re‐expressing CD45RA T cells, were significantly affected by ozanimod in our cohort. Although it is generally known that S1P_1_ ligands exert a stronger effect on certain lymphocyte subtypes, such as naïve T cells and central memory T cells expressing C–C chemokine receptor type 7 (CCR7),^31^ and minimal impact on NK cells,^28^, ^29^ this was not observed in our cohort. These discrepancies could be explained by the already pending lymphopenia in severe COVID‐19, and/or by the low S1P circulating levels,^4^, ^5^ resulting in already depleted circulating lymphocyte subpopulations.

Our results agree with the finding that patients with severe COVID‐19 feature an enrichment of classical monocytes in the circulation,^1^, ^32^ and we found that this population may be reduced in the OZA group compared with SOC. In patients receiving S1P receptor ligands for multiple sclerosis or Crohn's disease, ozanimod did not lower monocyte numbers even when administered during long periods and at higher doses than in our cohort.^28^, ^29^ Nevertheless, these studies were not performed under severe acute inflammation. The decrease in monocytes seen here is reminiscent of what was observed in animal influenza models using S1P receptor ligands,^6^ suggesting that S1P receptor ligands could differentially impact leukocyte populations under severe inflammation *versus* homeostasis or low‐grade chronic inflammation.

CCL2, a potent chemoattractant for monocytes^33^ strongly upregulated in COVID‐19,^34^ showed a blunted increase in the OZA group compared with the SOC group. As CCL2 levels were linked to circulating monocyte numbers under inflammatory conditions,^35^ and classical monocytes are the main monocyte subset expressing C–C chemokine receptor type 2 (CCR2), the CCL2 receptor,^36^, ^37^ it is plausible that ozanimod may alter the classical monocyte response by modulating CCL2. Knowing that classical monocytes comprise at least one subset associated with COVID‐19 severity,^32^, ^38^ it is tempting to speculate that such an effect may be beneficial. The CCL2–monocyte link has indeed been pointed out as a putative mechanistic target for treating COVID‐19 and acute respiratory distress syndrome^39^ and could explain, at least in part, the beneficial effects of S1P ligands as a treatment for severe forms of COVID‐19.^14^

Other cytokines such as IL‐8, IL‐6, interferon‐γ, CXCL10 and CXCL13 may be impacted by ozanimod, which aligns with the ability of S1P_1_ modulators to decrease inflammatory cytokines during viral pneumonia as seen in preclinical models.^6^ Interestingly, the possible decrease of IL‐8, an important chemokine for neutrophil attraction and recruitment, in the OZA group could explain why neutrophil circulating levels were shown to be lower in the OZA group than those in the SOC group in the COZI trial.^14^ Of interest, the cytokines highlighted as potentially impacted by ozanimod here were previously identified as key targets of S1P receptor ligands to prevent inflammation in experimentally induced viral pneumonia.^6^ While CCL2 levels showed a robust decline in the OZA group, other mediators identified in this study only showed trends, which sometimes were weak. Additional studies with larger cohorts are required to confirm or refute the impact of ozanimod on inflammatory mediators.

At the 90‐day follow‐up, vascular endothelial growth factor levels were higher in the OZA group compared with the SOC group, and other analytes also tended to be higher in the OZA group. Despite the observed differences, cytokine levels remained low overall, raising the question of whether these differences have a biological impact. While the data do not exclude the possibility of a delay in the repair or resolution process, the precise implications of these findings require further investigation. Our results suggest that transient ozanimod delivery may cause long‐term, yet mild, modifications of circulating cytokine levels, which may speculatively be attributed to a delay in healing.

Our results show that ozanimod therapy started during acute SARS‐CoV‐2 infection could impede the specific long‐term humoral immunity. This echoes the results obtained in patients receiving ozanimod as a disease‐modifying therapy for multiple sclerosis who show, for the most part, seroconversion, but a reduced specific SARS‐CoV‐2 antibody response.^10^, ^11^ Similarly, Boulton *et al*.^40^ showed that intradermal vaccination 1 week after the onset of fingolimod (an S1P_1–3,5_ receptor ligand prodrug) treatment leads to attenuated antibody responses. Whether ozanimod would also impair the humoral response in the context of an ongoing SARS‐CoV‐2 infection was uncertain, given that S1P receptor ligands typically do not compromise established humoral responses,^40^ and that ozanimod was administered for a relatively short duration (i.e. a maximum of 14 days).^14^ Here, the median duration of symptoms before enrollment and ozanimod administration was 10 days and the majority of patients had an ongoing specific anti–SARS‐CoV‐2 humoral response at treatment onset, which similarly increased for patients from the SOC or OZA group at day 5, suggesting that ozanimod did not impair the induction of the anti–SARS‐CoV‐2 response. S1P receptor ligands can alter dendritic cell activities,^41^ disrupt normal lymphocyte trafficking,^42^ and induce lymphocyte apoptosis in lymph nodes.^43^ Together, these could contribute to explaining the reduction of circulating SARS‐CoV‐2–specific antibodies on day 90 for unvaccinated patients for whom ozanimod was present during the primary infection. However, patients vaccinated after hospitalization but before follow‐up displayed robust anti–SARS‐CoV‐2 antibody levels on day 90. The robust hybrid immunity^44^ resulting from vaccination after infection suggests that vaccinated patients who received ozanimod are likely to benefit from a similar level of protection as their SOC counterparts. However, the precise nature of the Ig response and its potential link with antibody‐dependent cell‐mediated cytotoxicity after ozanimod treatment remains unknown, limiting our interpretation.

This study features obvious limitations, including its pilot nature, therefore, all analyses should be considered exploratory. With the security of S1P receptor modulators as treatment during severe viral infection now established,^13^, ^14^, ^45^ larger studies should be carried out to confirm the trends highlighted here. S1P receptor ligands were only tested in SARS‐Cov‐2 infections, and whether the mechanisms described here might be at play in severe pneumonia cases whose etiology differs remains to be determined. Nevertheless, the impact of S1P receptor ligands on CCL2 was established using influenza virus infection preclinical models,^6^, ^27^ and patients with a high risk of severe disease display low S1P levels in bacterial pneumonia,^46^ arguing that our findings may extend to different pneumonia etiologies. All analyses performed in this study are derived from blood tissue. While systemic inflammation is a good indication of the disease severity,^1^ the effects of ozanimod on local inflammation in the lungs for the treatment of severe viral pneumonia remain to be determined.

## CONCLUSION

Altogether, this study demonstrates that ozanimod administration during acute SARS‐CoV‐2 infection was associated with a reduction of circulating monocytes. Furthermore, we observed a blunted increase of circulating CCL2 and potentially of other mediators, such as IL‐8. Unvaccinated patients receiving ozanimod had lower levels of anti–SARS‐CoV‐2 IgGs on day 90 compared with the SOC group. However, vaccinated patients who received ozanimod during the acute phase of infection exhibited a robust anti–SARS‐CoV‐2 response. These findings suggest that ozanimod modulates key immune responses during acute SARS‐CoV‐2 infection, some of which align with mechanisms of action uncovered in murine infection models.

## METHODS

### Study design

The design of the COVID‐19 Intervention Study (COZI trial; NCT04405102) was approved by the Ethics Board (REB# MP‐10‐2021–3474) and conducted as described.^14^ In brief, the COZI trial was an open‐label, prospective, randomized and multicentric pilot trial conducted in the IUCPQ‐UL (Quebec Heart and Lung Institute, Quebec City, Canada), Cité‐de‐la‐Santé Hospital (CISSS de Laval, Canada) and Santa Cabrini Hospital (CIUSSS de l'Est‐de‐l'Ile‐de‐Montreal, Canada) from September 2020 to May 2022. Patients admitted for COVID‐19 who required oxygen support were eligible for the study. A total of 43 patients were recruited and separated into two groups: SOC and OZA. Randomization was stratified based on risk factors for poor outcomes and oxygen requirements at inclusion. The study design aimed to minimize systematic errors in group assignment at baseline.^14^ Participants in the OZA group received one 0.23‐mg ozanimod caplet for the first 4 days, then the daily dose was doubled (two 0.23‐mg caplets) and administered for a maximum of 10 days or until hospital discharge, whichever came first. The extensive inclusion and exclusion criteria are listed in the primary trial.^14^

### Differential counts, serum and peripheral blood mononuclear cell collection

Differential counts for monocytes were performed at the clinical laboratory. The serum sample was obtained by allowing the blood to clot in a serum‐separating tube for at least 30 min, followed by centrifugation at 1300 *g* for 10 min at room temperature. The serum sample was collected on days 1 (baseline; before the first ozanimod dose), 3, 5, 7, and during a follow‐up visit on day 90, and stored at −80°C. For peripheral blood mononuclear cell (PBMC) collection, 4 mL of ½ Hanks' balanced salt solution–diluted whole blood was poured on Ficoll and centrifuged at 400 *g* for 30 min without the brake. The portion containing PBMCs was then collected and washed two times using Hanks' balanced salt solution. PBMCs were frozen in Roswell Park Memorial Institute (RPMI) medium (Wisent, Saint‐Jean‐Baptiste, Canada) + 40% fetal bovine serum (Wisent, Saint‐Jean‐Baptiste, Canada) + 10% dimethylsulfoxide (Sigma‐Aldrich, St Louis, USA). Cells were kept for a maximum of 5 days at −80°C and then transferred in liquid nitrogen until use. PBMCs were collected on days 1, 3 and 7, and at follow‐up on day 90.

### Quantification of circulating analytes

Circulating analytes were measured via Luminex Assay. The serum sample was thawed and the concentration of 23 serum analytes was quantified using Human XL Cytokine Discovery Luminex (R) High‐Performance Assay (R&D Systems, Minneapolis, USA) according to the manufacturer's instructions. A solution of 4% formaldehyde (Sigma‐Aldrich, St Louis, USA) was added to the wells after the experiment, to deactivate the virus. Samples were read in duplicates with a Bio‐Plex MAGPIX (Bio‐Rad Laboratories, Hercules, USA).

### Flow cytometry

Peripheral blood mononuclear cells were rapidly thawed at 37°C and transferred into 20 mL of phosphate‐buffered saline. PBMCs were pelleted, resuspended in phosphate‐buffered saline and labeled with the Zombie Yellow viability dye (BioLegend, San Diego, USA) for 15 min at room temperature. Cell surface labeling was performed for 20 min at room temperature using antibodies listed in Supplementary table 1. Cells were then washed and fixed using 1% paraformaldehyde (Sigma‐Aldrich, SaintLouis, USA) for 1 h at 4°C before data acquisition. A healthy control volunteer without COVID‐19 was used to set fluorescence minus one control and to ensure interexperience consistency. Median fluorescence intensities were compiled after removing baseline fluorescence obtained with fluorescence minus one control. Measures were collected using a FACSDiva–driven customized LSR Fortessa (BD Biosciences, Franklin Lakes, USA) and analyzed using the FlowJo software (Tree Star, Woodburn, USA, Version 10.8.1).

### Anti–SARS‐CoV‐2 antibody measurement

Anti–SARS‐CoV‐2 QuantiVac ELISA kit (IgG; spike subunit 1) (EUROIMMUN, Lübeck, Germany) was used according to the manufacturer's guidelines at baseline (day 1), on day 5 and on day 90. Each serum sample was tested minimally in duplicates. The lowest limit of detection was set with negative controls from the manufacturer and validated with the serum of four patients sampled before the COVID‐19 pandemic (2018). Patients tested on days 1 and 5 were required to be previously unvaccinated and samples were available for both days. All patients were tested at follow‐up (i.e. day 90).

### Statistical analyses

The laboratory data were analyzed using a linear mixed model with one fixed factor for the comparison between groups and another fixed factor associated with variability in the 3 levels of measurements taken on days 1, 3 and 7. Day 90 was analyzed separately. An interaction term between the two fixed factors was added to the statistical model. Variability among patients was analyzed as a random effect. The dependence among residuals of repeated measurements from the same experimental unit was estimated with an unstructured covariance association. The normality hypothesis was verified using the Shapiro–Wilks test using residuals from the statistical model and transformed by Cholesky's metric. Brown and Forsythe's variation of Levene's test statistic was used to verify the homogeneity of variances. Many variables were log‐transformed to fulfill the normality and variance assumptions. However, most analyses were also adjusted for age, gender and length of stay to validate this assumption. At day 90, all variables were analyzed using a Student's *t*‐test with the same transformation if it had been applied for the linear mixed model, or a Mann–Whitney *U*‐test if data were not normal at day 90. Statistical significance was indicated with a two‐tailed *P*‐value < 0.05. Analyses were performed using SAS version 9.4 (SAS Institute Inc, Cary, USA).

## AUTHOR CONTRIBUTIONS

**Olivier Courtemanche:** Conceptualization; formal analysis; investigation; methodology; visualization; writing – original draft; writing – review and editing. **Pascale Blais‐Lecours:** Conceptualization; methodology; project administration; visualization; writing – review and editing. **Sylvie Lesage:** Formal analysis; resources; writing – review and editing. **Geneviève Chabot‐Roy:** Formal analysis; investigation; writing – review and editing. **Lise Coderre:** Formal analysis; investigation; writing – review and editing. **Marie‐Renée Blanchet:** Supervision; writing – review and editing. **Nathalie Châteauvert:** Conceptualization; writing – review and editing. **François Lellouche:** Conceptualization; funding acquisition; investigation; methodology; resources; writing – review and editing. **David Marsolais:** Conceptualization; funding acquisition; investigation; methodology; resources; supervision; visualization; writing – original draft; writing – review and editing.

## CONFLICT OF INTEREST

This trial was sponsored by IUCPQ‐ULaval. This study was partially funded by Bristol Myers Squibb (BMS), which provided the investigational medication. BMS had no decisional role in the study design, data collection, data analysis, data interpretation or writing of the report. BMS staff were provided with the manuscript before submission.

## Supporting information


Supplementary figure 1
Supplementary figure 2
Supplementary figure 3
Supplementary figure 4
Supplementary table 1
Supplementary table 2

## Data Availability

The data that support the findings of this study are available on request from the corresponding author. The data are not publicly available due to privacy or ethical restrictions.
