# Supplementary material for: Exploratory analyses of leukocyte responses in hospitalized patients treated with ozanimod following a severe acute respiratory syndrome coronavirus 2 (SARS‑CoV‑2) infection
Source: Immunol Cell Biol. 2025 Mar 2;103(5):433–43. doi: 10.1111/imcb.70006 (PMC12108697; doi:10.1111/imcb.70006)
Supplement: Supplementary file 1 — Supplementary figure 1 Supplementary figure 2 Supplementary figure 3 Supplementary figure 4 Supplementary table 1 Supplementary table 2 [file IMCB-103-433-s001.pdf]

Supporting information

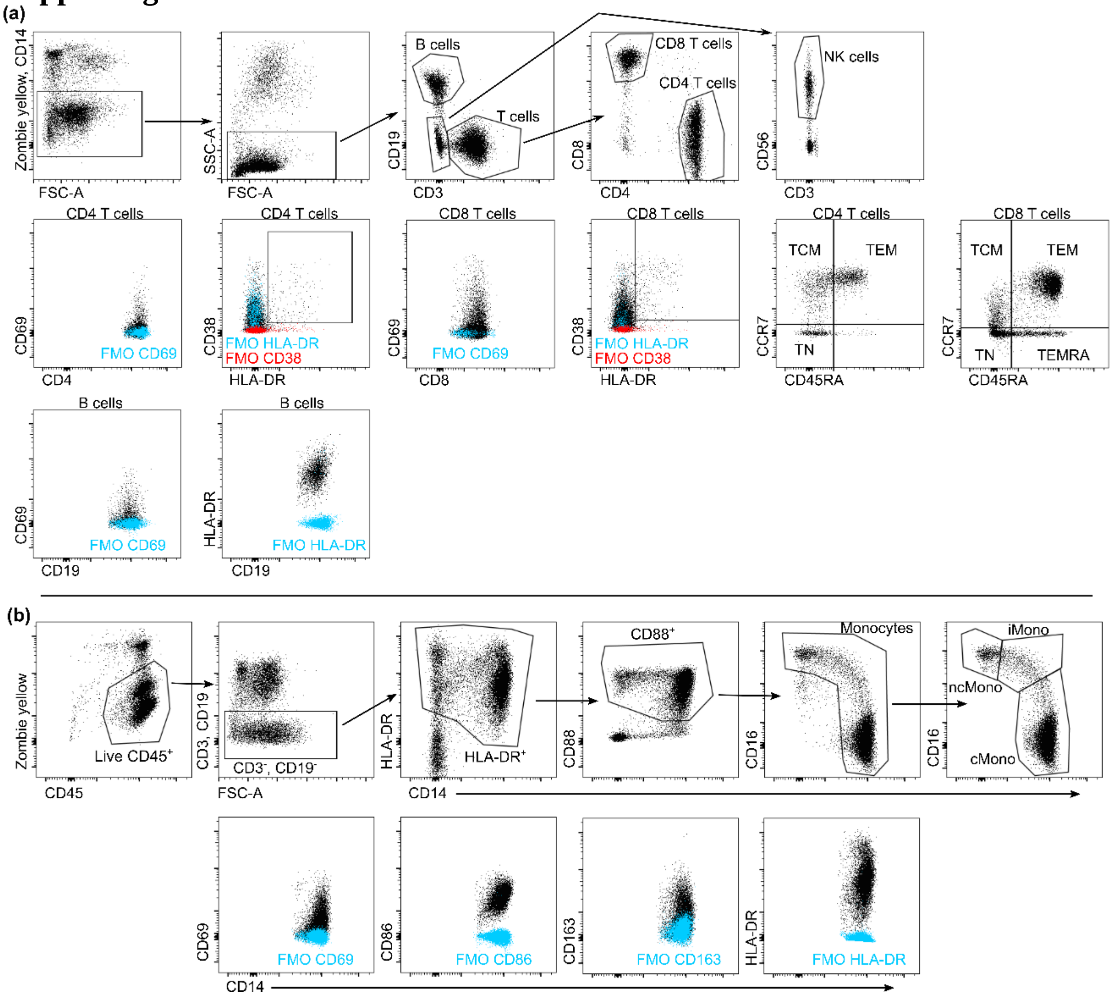

Supplementary figure 1: Flow cytometry gating on PBMC. PBMCs from severe COVID-19 patients were isolated for flow cytometry analyses of lymphocytes and monocytes. **(a)** Lymphocytes were gated using low FSC-A and SSC-A $CD14^-$ Zombie $Yellow^-$ cells. B cells were gated as $CD19^+CD3^-$ cells, while T cells were gated as $CD3^+CD19^-$ cells. NK cells were isolated as $CD19^-CD3^-CD56^+$ lymphocytes. CD4 and CD8 subsets were identified using CCR7 and CD45RA and activation markers (CD69, HLA-DR, CD38, and PD-1) were assessed on the different lymphocyte subpopulations. **(b)** Monocytes were gated as Zombie $yellow^-$ $CD3^-CD19^-HLA\text{-}DR^+CD88^+$ cells. Monocytes were classified as classical monocytes (cMono), intermediate monocytes (iMono), and non-classical monocytes (ncMono) based on their respective CD14 and CD16 expression. Activation/functional markers (CD69, CD86, CD163, and HLA-DR) were assessed on monocyte subsets. For both gating strategies, completely stained examples are shown in black, and fluorescence minus one (FMO) controls are shown in blue and red. NK cells, Natural Killer cells; TCM, Central memory T cells; TEM, Effector memory T cells; TN, Naïve T cells; TEMRA, Effector memory cells re-expressing CD45RA.

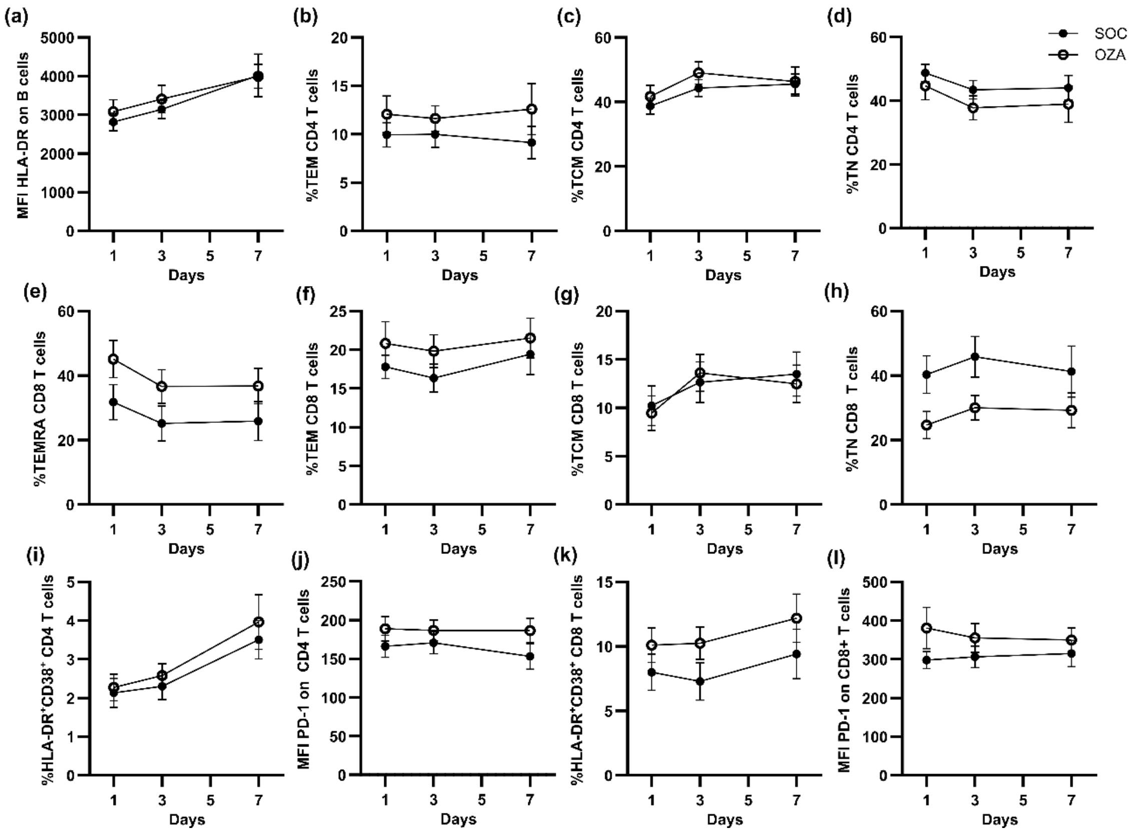

Supplementary figure 2: Lymphocyte activation markers and T cell subsets in the SOC and the OZA groups. Lymphocyte subsets were gated as described in Supplementary figure 1A. **(a)** The level of HLA-DR on B cells is shown. **(b-l)** The proportion of CD4 **(b-d)** and CD8 **(e-h)** T cell subsets and **(i-l)** the expression of activation markers on T cell subsets were quantified by flow cytometry. TCM, Central memory T cells; TEM, Effector memory T cells; TN, Naïve T cells; TEMRA, Effector memory cells re-expressing CD45RA.

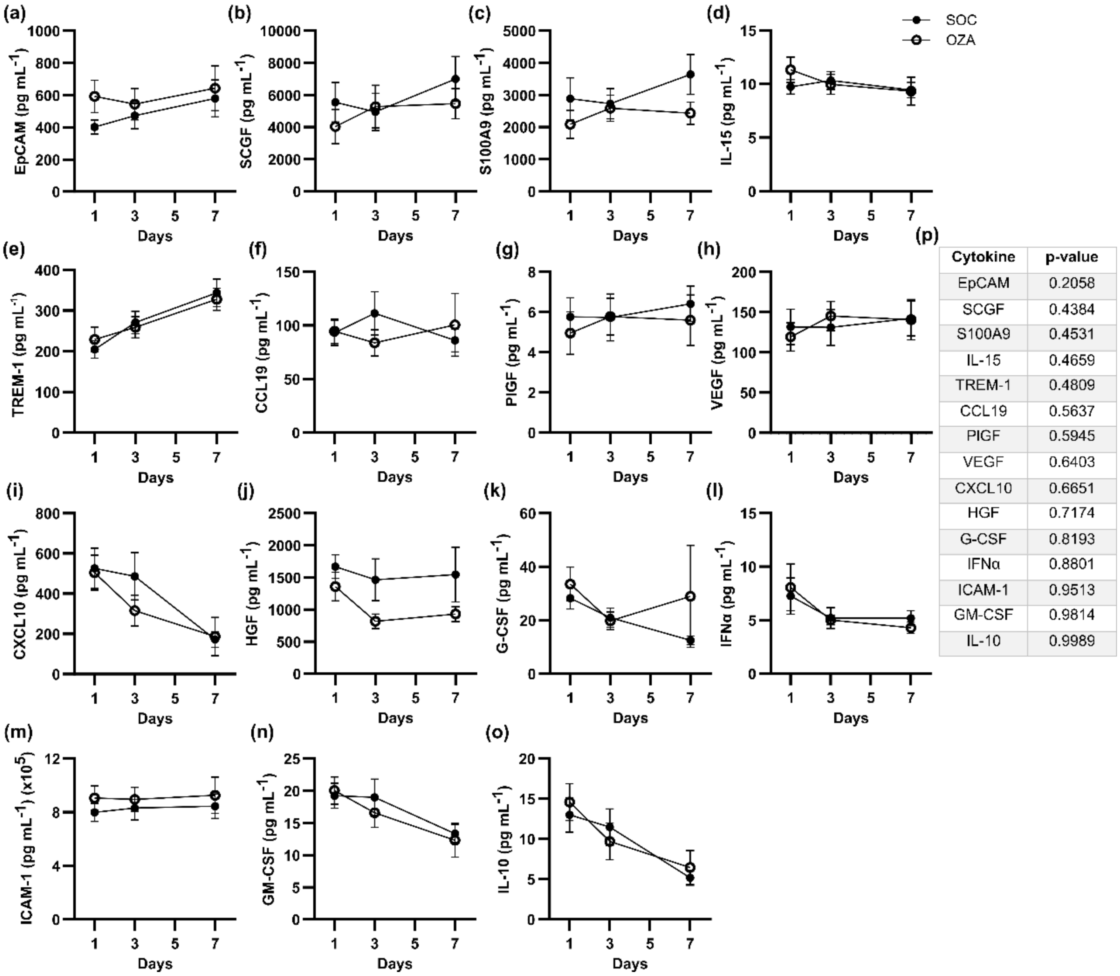

(p)

Cytokine	p-value
EpCAM	0.2058
SCGF	0.4384
S100A9	0.4531
IL-15	0.4659
TREM-1	0.4809
CCL19	0.5637
PIGF	0.5945
VEGF	0.6403
CXCL10	0.6651
HGF	0.7174
G-CSF	0.8193
IFNα	0.8801
ICAM-1	0.9513
GM-CSF	0.9814
IL-10	0.9989

Supplementary figure 3: Levels of circulating analytes in the acute phase of severe COVID-19. Serum was collected from patients in the SOC group and from the OZA group at baseline (day 1; before ozanimod administration) and during treatment (on days 3 and 7). **(a-o)** A Luminex assay was performed to evaluate the levels of various circulating analytes in both groups. **(p)** *P*-values for group-time interaction are indicated. Averages ± SEM are shown for each tested day in each group. n=11-22 for SOC and 11-18 for OZA.

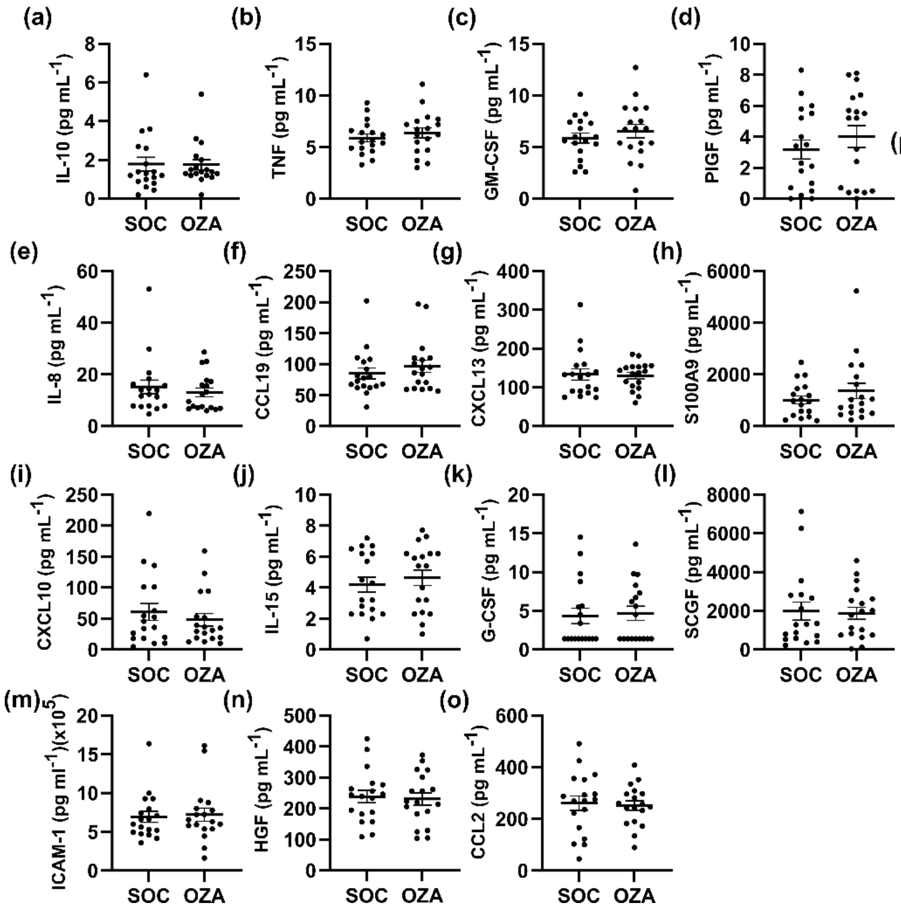

(p)

Cytokine	p-value
IL-10	0.4189
TNF	0.4334
GM-CSF	0.4350
PIGF	0.4567
IL-8	0.5010
CCL19	0.5161
CXCL13	0.5418
S100A9	0.5628
CXCL10	0.6340
IL-15	0.6436
G-CSF	0.7030
SCGF	0.7666
ICAM-1	0.7666
HGF	0.7779
CCL2	0.7964

Supplementary figure 4: Levels of circulating analytes at follow-up. Serum was collected at follow-up (day 90 post entry). **(a-o)** A Luminex assay was performed on serum to evaluate the levels of various circulating analytes in both groups. **(p)** *P*-values for group-time interaction are indicated. Averages ± SEM are shown for each tested day in each group. n=18 for both groups.

Supplementary table 1: Antibodies used for flow cytometry

**Antibody**	**Clone**	**Company (Catalog number)**
CD14- BV570	M5E2	BioLegend (301832)
CD3- AF700	UCHT1	BD (557943)
CD19- APC	HIB19	BioLegend (302212)
CD4- BV711	SK3	BD (563028)
CD8- BV786	HIT8a	BD (740988)
CD45RA- PerCP	HI100	BioLegend (304156)
CCR7- PE-Dazzle594	G043H7	BioLegend (353236)
CD38- FITC	HIT2	BD (555459)
HLA-DR- APC-Cy7	L243	BioLegend (307618)
PD-1- PE-Cy7	EH12.1	BD (561272)
CD56- BV421	5.1H11	BioLegend (362552)
CD69- PE	FN50	BioLegend (310906)
CD3- BV711	UCHT1	BioLegend (300464)
CD19- BV711	HIB19	BD (740774)
CD88- FITC	S5/1	BioLegend (344306)
CD45- APC-Cy7	HI30	BioLegend (304014)
CD14- AF700	63D3	BioLegend (367114)
CD16- BV786	3G8	BD (563690)
HLA-DR- PB	L243	BioLegend (307633)
CD86- PE-Dazzle594	IT2.2	BioLegend (305434)
CD11c- PerCP	Bu15	BioLegend (337234)
CD163- PE-Cy7	GHI/61	BioLegend (333614)

Supplementary table 2: Vaccination status for patients in Figure 5b

	**SOC**	**OZA**
**Unvaccinated**	7	6
**Vaccinated before randomization**	2	2
**Vaccinated between randomization and follow-up**	5	9
**Unknown vaccination status**	4	1
